# Analysis of 6-week mortality and influencing factors in patients with liver cirrhosis and portal vein thrombosis complicated by acute gastrointestinal bleeding

**DOI:** 10.1371/journal.pone.0345079

**Published:** 2026-03-20

**Authors:** Junli Zhang, Ling Feng

**Affiliations:** 1 Department of Gastroenterology and Hepatology, West China Hospital, Sichuan University/West China School of Nursing, Sichuan University, Chengdu, PR China; 2 Department of Neurology, West China Hospital, Sichuan University/West China School of Nursing, Sichuan University, Chengdu, PR China; Dalin Tzu Chi Hospital, Buddhist Tzu Chi Medical Foundation, TAIWAN

## Abstract

**Objective:**

To explore the 6-week mortality of acute gastrointestinal bleeding in patients with liver cirrhosis and portal vein thrombosis and to analyze its influencing factors.

**Methods:**

This was a retrospective study. We retrospectively screened 232 patients with liver cirrhosis and portal vein thrombosis complicated by acute gastrointestinal bleeding who were admitted to West China Hospital of Sichuan University from January 1，2020 to November30，2022. Of the 232 patients, 75 had their first bleeding. Additionally, the patients were divided into a mortality group (n = 34) and a non-mortality group (n = 198) based on whether they died within 6 weeks of the onset of gastrointestinal bleeding. Baseline general information, laboratory indicators, and other clinical data of the two groups were compared, and the independent risk factors for 6-week mortality in patients with liver cirrhosis and portal vein thrombosis complicated by acute gastrointestinal bleeding were analyzed.

**Results:**

There were significant differences between the two groups in terms of the etiology of liver cirrhosis, HCC, 5-day treatment failure rate (8.62%), TBiL, ALT, BUN, Na, eGFR, DBiL, AST, GGT, ALP, CR, WBC, PLT, and PT(p < 0.05). Multivariate logistic regression analysis revealed that elevated ALP levels showed a positive association with 6-week mortality [OR=1.01, 95% CI (1.01, 1.01), p < 0.01] and 5-day treatment failure showed a positive association with 6-week mortality [OR=11.27, 95% CI (3.45, 36.81),p < 0.01].

**Conclusion:**

Elevated ALP and 5-day treatment failure were found to be independent risk factors for 6-week mortality in patients with cirrhosis and portal vein thrombosis complicated by acute gastrointestinal bleeding.

## Introduction

Cirrhosis was a chronic disease characterized by hepatocyte necrosis, regenerative nodule formation, and liver tissue fibrosis [1], and was the 11th most common cause of mortality worldwide, accounting for 3.5% of global deaths [2]. During the decompensated stage of liver cirrhosis, various complications, such as portal vein thrombosis (PVT), ascites and gastrointestinal bleeding, could occur. PVT referred to the formation of blood clots in the main trunk and branches of the portal vein, which could extend to the splenic or mesenteric veins, causing complete or partial obstruction of the lumen [3]. PVT was a common complication of liver cirrhosis, but its onset in patients with liver cirrhosis was insidious and difficult to detect in the early stage of disease. The pathogenesis of PVT in patients with cirrhosis was likely multifactorial and resulted mainly from alterations in the different components of Virchow’s triad: decreased portal vein flow, hypercoagulability and endothelial injury [4]. The incidence of PVT in patients with decompensated liver cirrhosis was 0.6% to 26% [5]. Patients with cirrhosis and PVT were prone to various complications, such as ascites and gastrointestinal bleeding [6].

Acute gastrointestinal bleeding (AGIB) in this study specifically referred to bleeding caused by underlying diseases (liver cirrhosis and PVT), which included rupture of esophagogastric varices, portal hypertensive gastropathy,while excluding other types of bleeding. Gastrointestinal bleeding was a critical and dangerous complication of liver cirrhosis, with a high mortality rate of approximately 11.1% [7]. The incidence of acute upper gastrointestinal bleeding in patients with liver cirrhosis was 5% to 15% annually, and the mortality rate associated with variceal bleeding was 7% to 20% [8]. According to the latest Baveno VII consensus [9], 6-week mortality should be considered the primary endpoint of treatment studies for acute variceal bleeding in patients with liver cirrhosis. Six-week mortality was defined as death within 6 weeks after the onset of acute gastrointestinal bleeding. Five-day treatment failure was defined as uncontrolled bleeding or recurrent bleeding within 5 days. PVT could lead to a further increase in portal vein pressure, making esophageal and gastric varices more serious, and increasing the risk of bleeding, seriously threatened the life of patients.At present, there were few studies on the risk factors for 6-week death in patients with liver cirrhosis and portal vein thrombosis complicated by acute gastrointestinal bleeding.To investigate the risk factors of 6-week death in patients with liver cirrhosis and portal vein thrombosis complicated by acute gastrointestinal bleeding was of great significance for early identification of high-risk patients, formulation of individualized treatment plan and improvement of patient prognosis.The purpose of this study was to retrospectively analyze the clinical data of patients with liver cirrhosis and portal vein thrombosis complicated by acute gastrointestinal bleeding, to explore the risk factors affecting the 6-week death of patients, to provide a reference for clinicians, and to provide a theoretical basis for improving the prognosis of patients.

## Materials and methods

### Study subjects

This was a retrospective study.Retrospective data were continuously collected from hospitalized patients who were diagnosed with cirrhosis-related PVT complicated with acute gastrointestinal bleeding at West China Hospital of Sichuan University between January 1，2020 and November 30， 2022. Of the 232 people, 75 had their first bleeding. These patients’ information (name, gender, age, etc.) was available through the hospital’s electronic medical record system. And 6-week mortality was evaluated as the primary outcome for patients with liver cirrhosis and portal vein thrombosis complicated by acute upper gastrointestinal bleeding. Patients were divided into a mortality group (n = 34) and a non-mortality group (n = 198). The inclusion criteria [10–12]: (1) patients met the diagnostic criteria for liver cirrhosis(clinical presentation, laboratory tests, imaging examinations, or liver biopsies); (2) PVT was diagnosed through Doppler ultrasound, enhanced CT, or magnetic resonance imaging (MRI) [13–15]: Imaging studies revealed filling defects and blood flow interruption in the main portal vein and (or) its branches.Doppler ultrasonography was the preferred initial imaging modality, while contrast-enhanced CT and MRI provide higher diagnostic accuracy for PVT in cirrhotic patients, with additional capability to delineate thrombus extent; (3) AGIB related to liver cirrhosis and PVT confirmed by endoscopy or imaging (excluding non-portal hypertension-related bleeding); (4) Age ≥ 18 years old. The exclusion criteria were as follows: (1) cases with incomplete data; (2) patients with severe cardiovascular or pulmonary diseases.

### Data collection

General information, including age, sex, and etiology of liver cirrhosis, was collected. The laboratory indicators collected included: white blood cell (WBC) count; hemoglobin (Hb) count; platelet (PLT) count; prothrombin time (PT); international normalized ratio (INR); total bilirubin (TBiL); direct bilirubin (DBiL); indirect bilirubin (IBiL); alanine aminotransferase (ALT); aspartate aminotransferase (AST); alkaline phosphatase (ALP); glutamyl transpeptidase (GGT); albumin (ALB); serum creatinine (Scr); blood urea nitrogen (BUN); glucose (GLU); creatinine (CR); and glomerular filtration rate (eGFR). Complications included ascites, hepatic encephalopathy, and HCC were noted,and Child‒Pugh score of the patient was calculated. The treatment effect was observed within 5 days and followed up for six weeks to observe mortality.

### Ethical statement

Because this was a retrospective study and data anonymization, so the study was conducted in accordance with the Declaration of Helsinki and approved by the Biomedical Ethics Review Committee, West China Hospital, Sichuan University for the waiver of informed consent, Number: 2023 (1446).

### Statistical analysis

SPSS 20.0 software was used to conduct the statistical analysis. Independent t-test was used to compare age and ALP between the two groups. The results of laboratory examination between the two groups were compared by Mann -whitney U test. χ^2^ tests were used to compare the sex, etiology of cirrhosis, hepatic encephalopathy and ascites between the two groups. Based on clinical relevance and check collinearity (VIF, threshold < 5), statistically significant variables identified in the univariate analysis were included in the multivariate analysis.. Multiple logistic regression analysis was used to explore the influencing factors of 6-week outcomes in patients with PVT complicated by gastrointestinal bleeding in liver cirrhosis patients. Differences were considered statistically significant at p < 0.05.

## Results

A total of 232 patients with liver cirrhosis and portal vein thrombosis complicated by acute gastrointestinal bleeding, including 174 males (75%) and 58 females (25%), were included in this study. One hundred and sixty-four patients (70.69%) had viral hepatitis, 134 patients (57.76%) had a grade B Child‒Pugh score, 102 had Hepatocellular carcinoma (HCC), and 20 patients experienced five-day treatment failure. Thirty-four patients died within 6 weeks, for a mortality rate of 14.65%. The average age of the 6-week mortality group was 57.62 ± 12.52 years, while the average age of the 6-week non-mortality group was 54.94 ± 11.82 years. In the 232 cases,there was a statistically significant difference in the etiology, incidence of HCC, and incidence of 5-day treatment failure between the two groups (p < 0.05), while there was no statistically significant difference in age, sex, hepatic encephalopathy, or ascites (p > 0.05), as shown in Table 1.

**Table 1:** Univariate analysis of the 6-week mortality group and non-mortality group (general information and complications).

Variables	6-week mortality group (n = 34)	6-week non-mortality group(n = 198)	p Value
**Gender**			
**Male**	26(76.5%)	148(74.6%)	0.830
**Female**	8(23.5%)	50(25.2%)	
**Age**	57.62 ± 12.52	54.94 ± 11.82	0.228
**Etiology of liver cirrhosis**			
**Viral hepatitis**	29(85.3%)	135(68.2%)	0.043
**Non-viral hepatitis**	5(14.7%)	63(31.8%)	
**Child‒Pugh score**			
**A**	2(5.9%)	50(25.3%)	<0.001
**B**	17(50.00%)	117(59.1%)	
**C**	15(44.1%)	31(15.7%)	
**Hepatic encephalopathy**	7(18.9%)	24(12.1%)	0.180
**Ascites**	31(91.2%)	158(77.8%)	0.115
**HCC**	26(76.5%)	76(38.4%)	<0.001
**Bleeding history**			
**0**	13(38.2%)	62(31.3%)	0.425
**≥1**	21(61.8%)	136(68.7%)	
**5-day treatment failure**	11(32.4%)	9(4.6%)	<0.001

Laboratory examination revealed statistically significant differences (p < 0.05) in the TBiL, ALT, BUN, Na, eGFR, DBiL, AST, GGT, ALP, CR, WBC, PLT, and PT between the two groups, as shown in Table 2.

**Table 2:** Univariate analysis of 6-week mortality and non-mortality groups (laboratory indicators).

Variables	6-week mortality group(n = 34)	6-week non-mortality group(n = 198)	p Value
**TBiL, μmol/L**	33.1(21.35,56.28)	20.95(15.70,31.58)	0.001
**DBiL, μmol/L**	21.9(10.68,39,18)	9.4(6.56-15.20)	<0.001
**IBiL, μmol/L**	11.25(7.48,19.5)	10.85(7.8,16.14)	0.677
**ALT, IU/L**	47(24.75,79.00)	22(14.00,40.00)	<0.001
**BUN, mmol/L**	9.30(6.58,13.70)	6.8(6.50,10.35)	0.003
**Na, mmol/L**	135.70(132.2,137.55)	136.8(134.35,139.3)	0.034
**_eGFR, ml/min**	85.57(69.38,101.57)	97.29(82.95,109.01)	0.022
**AST, IU/L**	92.5(45.75,228)	33.5(23,56.25)	<0.001
**GGT, IU/L**	128.5(55,314.75)	43(22.75,90)	<0.001
**ALP, IU/L**	239.5(114.25,355.75)	82.5(60.75,135.25)	<0.001
**GLU, mmol/L**	7.71(6,11.03)	7.02(5.76,9.21)	0.302
**CR, μmol/L**	79.5(66,98.25)	71(60,84.25)	0.039
**Hb, g/L**	82.5(61.5,94.00)	72(61.75,86.25)	0.269
**WBC, × 10** ^**9**^ **/L**	6.26(4.82,11.50)	4.64(3.01,7.04)	0.002
**PT, s**	15(13.98,16.83)	14(13,15.5)	0.010
**PLT, × 10** ^**9**^ **/L**	95(58.5,152.5)	64.5(40,108.25)	0.008
**INR**	1.36(1.26-1.48)	1.28(1.18,1.41)	0.057
**ALB,g/L**	29.59 ± 5.73	30.50 ± 3.93	0.377

With 6-week mortality as the dependent variable (mortality: 1, non-mortality: 0), Based on clinical relevance,variables with statistical significance in univariate analysis were included in multivariate analysis after excluding collinear factors. Categorical variable assignment: 5-day treatment failure (Yes: 1, No: 0). Multivariate logistic regression analysis revealed that ALP and 5-day treatment failure were risk factors for 6-week mortality in patients with liver cirrhosis and portal vein thrombosis complicated by acute gastrointestinal bleeding (p < 0.01), as shown in Table 3. Model diagnostics indicated no significant collinearity (all VIF < 5), good calibration (Hosmer-Lemeshow test p = 0.52), and acceptable discrimination (C-statistic = 0.79).

**Table 3:** Multivariate logistic regression analysis of the 6-week mortality group and non-mortality group.

Variables	B	SE	Wald	pValue	OR Value	95% CI
**ALP**	0.01	0	21.41	<0.001	1.01	1.01,1.01
**5-day treatment failure**	2.42	0.60	16.09	<0.001	11.27	3.45,36.81
**Child‒Pugh score(B)**	1.21	0.85	2.04	0.153	3.36	0.64,17.77
**Child‒Pugh score(C)**	1.98	0.9	4.88	0.057	7.23	0.95,21.84

Among the 34 deceased patients, the distribution of causes of death was as follows: rebleeding (17 cases, 50.00%), liver failure (9 cases, 26.47%), multiorgan failure (4 cases, 11.76%), ther causes (3 cases, 8.82%), and sepsis (1 cases, 2.94%).

## Discussion

At present, some studies had investigated 6-week outcomes of patients with liver cirrhosis and PVT who suffered from gastrointestinal bleeding, but there were different risk factors in different studies.This study examined the risk factors for 6-week mortality in patients with liver cirrhosis and portal vein thrombosis complicated by acute gastrointestinal bleeding, providing a clinical reference. Early intervention in these influencing factors might improve patient prognosis and increase patient survival. This study retrospectively investigated 232 cases of PVT complicated with acute gastrointestinal bleeding in patients with liver cirrhosis, 34 of which died at 6 weeks. The insidious onset of PVT in patients with liver cirrhosis was usually detected during routine examinations or other liver cirrhosis examinations in asymptomatic patients [16], which mainly affected the prognosis of patients with poor liver function. Some studies had shown that PVT represented a significant and serious complication in patients with liver cirrhosis and was considered to be a negative prognostic factor in patients with cirrhosis [17,18,19]. PVT was more common in patients with poorer liver function, and the higher the Child‒Pugh liver function grade was, the greater the risk of rebleeding and death [20]. In this study, 146 patients (52.3%) with acute gastrointestinal bleeding caused by PVT and liver cirrhosis had Child‒Pugh score of grade B for liver function, and the ALP level in the mortality group was greater than that in the non-mortality group.

Late-stage liver damage caused by various factors could develop into cirrhosis, with the main clinical manifestations of cirrhosis being liver dysfunction and portal hypertension. The liver was the site for the synthesis of various coagulation factors, and impaired liver function could affect the secretion and synthesis of coagulation fibrinolytic factors, as well as the liver’s ability to clear tissue plasminogen activator substances, ultimately putting the blood in a hypercoagulable state and easily inducing PVT. PVT increased portal vein pressure, which might trigger bleeding events related to portal vein hypertension and even death [21]. The causes of upper gastrointestinal bleeding in patients with liver cirrhosis included the rupture of esophageal and gastric varices, the presence of peptic ulcers, portal hypertensive gastropathy, and gastric vasodilation [22, 23], among which the most common cause was the rupture of esophageal and gastric varices. Damage to the varicose vein wall or a sudden increase in portal vein pressure caused by various factors could lead to esophageal variceal rupture and bleeding, posing a serious threat to the patient’s life. When liver cirrhosis was complicated with PVT, thrombotic obstruction led to increased resistance to portal venous outflow, which elevated portal pressure; this elevated pressure subsequently drove the formation of collateral circulation and the dilation of varices, thereby increasing the risk of rupture and bleeding.. The development of this disease ultimately led to esophageal and gastric varicose vein rupture and bleeding. Therefore, gastrointestinal bleeding in liver cirrhosis and the PVT interacted with each other [24]. Multiple studies had shown [6, 25, 26] that patients with cirrhosis combined with PVT had a greater risk of gastrointestinal bleeding and mortality than those without PVT.

### Comparison with Previous Studies

This study found that the 6-week mortality rate of patients with liver cirrhosis and PVT complicated by AGIB was 14.66%, which was consistent with the previously reported range of 15%−20% [8,24]. This consistency confirmed the reliability of our results, while the slight difference might have been to variations in sample size and patient characteristics.

Earlier studies identified hepatic encephalopathy, Hb, C-reactive protein, PT, and TBiL as independent risk factors for 6-week mortality in cirrhotic patients with AGIB [27]. However, this study did not include these indicators in the final model, which might have been related to the specific population (only patients with PVT) and model optimization (excluding collinear variables). Rahul et al. [28] emphasized that the severity of acute-on-chronic liver failure (ACLF) affects mortality in cirrhotic patients with variceal bleeding, but ACLF was not a focus of this study. Xuefeng Luo et al. [29] reported that 5-day treatment failure is an independent risk factor for 6-week mortality in cirrhotic patients with portal cavernoma, which was consistent with our findings. In this study, 55% (11/20) of patients with 5-day treatment failure died, indicating that recurrent or uncontrolled bleeding was a key predictor of poor prognosis. Preventing rebleeding within 5 days might have effectively reduced mortality.

ALP elevation was identified as a new independent risk factor in this study, which differed from previous studies [27,30]. This might have been because this study focused on patients with PVT: elevated ALP reflected cholestasis in cirrhotic patients with PVT [31], which exacerbated liver injury, impaired response to thrombotic therapy, and increased the risk of complications and mortality. A retrospective study [32] predicting the risk of variceal rehemorrhage in cirrhotic patients with portal vein thrombosis was shown that C-reactive protein (p < 0.001), and AST (p = 0.039) were independently associated with variceal rehemorrhage,not mention of ALP. This might be due to different patient characteristics and follow-up time. Zuo et al. [33] also found that ALP was significantly higher in patients with severe liver injury, supporting our result.

### Mechanisms Underlying Risk Factors

Elevated ALP: ALP was widely distributed in the liver, bones, and biliary system, and its elevation in cirrhotic patients with PVT was mainly related to cholestasis [31]. Vincenza Calvaruso et al.‘s study [34] showed portal pressure correlated significantly with a semiquantitative grading of cholestasis.The Expert Consensus for the Management of Portal Vein Thrombosis in Liver Cirrhosis [35] indicated that PVT was associated with the severity of hepatic impairment. As liver reserve function declined and hepatic function deteriorated, the prevalence and cumulative incidence of PVT significantly increased, adversely affecting the prognosis of cirrhotic patients—particularly those with more advanced liver dysfunction. This suggested an indirect link between liver function markers (such as ALP) and PVT severity, implying that hepatic function indicators could, to some extent, reflect both the severity of PVT and patient outcomes.Elevated ALP levels reflected cholestasis in cirrhotic patients with PVT. Cholestasis exacerbated liver injury, predisposed patients to complications and impaired response to thrombotic therapy, ultimately increasing mortality risk. Although this study did not directly validate the aforementioned mechanisms, existing evidence and cohort characteristics supported this logical association. Future research would incorporate more in-depth mechanistic exploration and dynamic data collection to further substantiate these findings.

5-day treatment failure: Recurrent or uncontrolled bleeding within 5 days indicated ineffective hemostasis or severe underlying liver function impairment. PVT-induced portal hypertension increased the difficulty of hemostasis, and repeated bleeding could lead to hypovolemic shock, hepatic encephalopathy, and other complications, ultimately increasing mortality [36].

The cause-of-death distribution revealed a clear hierarchy of fatal risks in patients with cirrhosis and PVT complicated by AGIB, with rebleeding (50.00%) and liver failure (26.47%) accounting for over three-quarters of deaths. This finding directly validated the study’s core risk factor conclusions and provided actionable clinical insights.

Rebleeding’s predominance (50.00%) reflected the unique pathophysiological burden of this cohort: PVT-induced portal venous outflow obstruction sustained portal hypertension, rendering esophagogastric varices fragile even after initial hemostasis [6,21]. Fluctuations in portal pressure, cirrhosis-related coagulopathy, or inadequate initial treatment easily triggered recurrent bleeding, which induced hypovolemic shock and hepatic ischemia-hypoxia—exacerbating liver dysfunction and forming a fatal vicious cycle. This explained why 5-day treatment failure emerged as a strong independent risk factor (OR=18.32), emphasizing that early, durable hemostasis (e.g., endoscopic variceal ligation) and proactive rebleeding prophylaxis were the most impactful interventions to reduce mortality.

### Strengths and Limitations

Strengths: This study focused on the specific population of cirrhotic patients with PVT complicated by AGIB, optimized the statistical model to address overfitting, and supplemented cause-of-death data, providing targeted clinical evidence. ALP and 5-day treatment failure are easily measurable in clinical practice, facilitating early risk stratification.

Limitations: Although this study provides new insights into the risk factors for 6-week death in patients with liver cirrhosis and portal vein thrombosis complicated by acute gastrointestinal bleeding some limitations remain. First, this study was a retrospective study. Despite our best efforts to collect complete data and conduct quality control, there might still be confounding factors that were not identified and controlled. Second, this study was a single center study, and the extrapolation of the findings might be limited. There might be differences in the characteristics of patients, the level of diagnosis and treatment and the treatment plan in different medical centers, and future multi-center studies were needed to further verify the results of this study.Third,mortality was only followed for 6 weeks and longer term outcomes could not be assessed.

### Clinical Implications

This study confirmed that elevated ALP and 5-day treatment failure are independent risk factors for 6-week mortality in patients with liver cirrhosis and PVT complicated by AGIB. Clinicians should closely monitor ALP levels and treatment response within 5 days in such patients. For those with elevated ALP, active intervention for cholestasis might be beneficial; for patients with 5-day treatment failure, timely adjustment of treatment strategies (e.g., endoscopic hemostasis or transjugular intrahepatic portosystemic shunt) might improve prognosis.

## Conclusion

This study identified elevated ALP and 5-day treatment failure as independent risk factors for 6-week mortality in patients with liver cirrhosis and PVT complicated by AGIB. These two readily measurable indicators provided reliable markers for clinical risk stratification of this specific population, and targeted interventions based on them could help optimize individualized treatment and improve short-term prognosis.

This study had inherent limitations. As a single-center retrospective study, it was subject to selection bias, which limited the generalizability of the findings. Additionally, the 6-week follow-up only reflected short-term mortality, and long-term prognostic outcomes could not be evaluated. The underlying mechanism linking elevated ALP to increased mortality was also not further verified by experimental studies.

Future research should conduct multi-center prospective studies to validate the current results with a larger sample size. Extending the follow-up duration will help clarify the long-term prognostic value of ALP and early treatment failure in this patient group. Furthermore, in-depth basic and clinical studies are needed to explore the specific mechanism of ALP elevation in aggravating poor outcomes of cirrhotic patients with PVT and AGIB, so as to provide a theoretical basis for developing new targeted therapeutic strategies.

## Supporting information

S1 Data Clinical characteristics and laboratory indicators data for all study participants. (XLSX)
